# God's Hand

**Published:** 1890-08-09

**Authors:** 


### Words of Consolation.

### GOD'S HAND.

"Thou hast beset me behind and before, and hast laid Thine hand upon me," says David in the 139th Psalm, and then he goes on to mention all that God has done for him in his creation and preservation, and exclaims, "Such knowledge is too wonderful, I cannot attain to it." In times of trouble the same David complains that God's hand was heavy upon him, and prays that the Lord will lay to His hand.

The Almighty acts by all His children in the same way now. When they are rebellious He chastens them in His wrath; when they will not recognise His correction He bears heavily upon them; if they cannot feel that it is His hand which is restraining their perverse and wandering steps, He shows them by the lightning of His power that he is Lord of all. But to the weary, the sick, the sorrowful, He comes in mercy, and it is in this light we will look on His dealings with us now.

We have all seen one of the saddest sights imaginable, a little child in a passion. He has wanted to do something that was wrong or would be hurtful to him, and his wilfulness and disobedience make him resist all interference. He cries, he stamps, he fights against those who would prevent him. Just as he is at the worst, his mother lays her firm gentle hand on him. Oh! the magic of the touch! He unwittingly owns the authority of one who speaks only to be obeyed. His struggles cease, his cries are hushed, and he falls sobbing and repentantly into the loving arms held out for his pardon and comfort.

How often are we like this little child. We vehemently long for a wider field of action, we chafe against the restraints which hold us, we seem to have more than we can bear in the opposition of those who should help and comfort us in our daily path. Do we feel these trials come from God's hand, and is His light and gentle touch to recall us to a sense of His presence and to a dependence on His will? or do we turn again and rend ourselves and others in the conflict? Do we make our moan and bewail our fate, saying no one ever had so much to put up with as ourselves? We can only hope that at such a time God will lay His hand on us and say, "Peace, be still." Oh! the bliss and the calm of that restraining grasp! the happiness of knowing that one mightier than ourselves has taken our cause into His keeping! We have been angry, disobedient, and rebellious, but we can now lie quiet and still in the Everlasting Arms, if we have the child-like spirit which owns, even in rebellion, the authority of the Parent.

And as the Good Physician, too, He heals by His touch. Are we sick? His touch soothes the ravings of delirium and the restless tossings of fever. Are we blind to our own good and bruise ourselves in groping blindly on? He opens our eyes by His hand. Are we morally covered with wounds and sores? He can, and will lay His hand upon us and heal us. Are we beside ourselves with passion or lust or vanity or pride? He only can cure us, for that sort of possession can be cast out entirely by prayer and fasting.

